# Effectiveness of a health literacy intervention targeting kidney patients and professionals: Marco Boonstra

**DOI:** 10.1093/eurpub/ckac129.637

**Published:** 2022-10-25

**Authors:** MD Boonstra, MS Gurgel do Amaral, GJ Navis, ME Stegmann, R Westerhuis, AF de Winter, SA Reijneveld

**Affiliations:** 1 Department of Health Sciences, University Medical Center Groningen, Groningen, Netherlands; 2 Department of Nephrology, University Medical Center Groningen, Groningen, Netherlands; 3 Department of General Practice and Elderly Cawre, University Medical Center Groningen, Groningen, Netherlands

## Abstract

**Background:**

Chronic kidney disease (CKD) patients with limited health literacy (LHL) experience a faster kidney decline. To counteract this, we developed Grip on your Kidneys (GoyK). This intervention targets patients’ communication and self-management. It trains health care professionals (HCPs) competences to support patients with LHL. This study aims to test the effectiveness of GoyK on patients’ health and self-management, HCPs’ communication competences, and the quality of consultations.

**Methods:**

A clustered and non-blinded quasi-experimental study was conducted, including 161 patients with mild to severe CKD and 48 HCPs from Dutch general practices and nephrology clinics. Patients (n = 77) and HCPs (n = 30) in the intervention group received GoyK. In the control group, patients (n = 76) had routine visits with HCPs (n = 19). Between March 2021 and June 2022, data were collected with questionnaires and from patient records at baseline (T0), 4 months (T1) and 9 months (T2). Primary outcomes were patients’ self-management and HCPs’ use of health literacy communication strategies.

**Preliminary results:**

At T1, the intervention improved the days per week patients exercised (B = 1.00, 95% confidence interval, CI = 0.35-1.65, P = 0.003), and Likert-scale reported (1-4) fluid intake (B = 0.37, CI = 0.10-0.63, p = 0.006). The intervention had a positive effect on several outcomes related to how patients perceived the consultation quality, and improved the reported use of communication strategies by HCPs at T1 (B = 0.68, CI = 0.35-1.01, p = <0.001). We found no effects on other patient outcomes, like activation for self-management or salt intake.

**Conclusions:**

Our health literacy intervention, targeting CKD patients with LHL and HCPs, improved lifestyle behaviors of patients and the quality of consultations. A further strengthening of other self-management behaviors and on HCPs’ competences is needed, also to reach sustainable effects in the care for patients with LHL.

**Key messages:**

• A health literacy intervention, targeting patients and professionals simultaneously, improved the patients’ self-management and care consultations.

• Training of HCPs improved their competences to support patients with LHL, and care organizations and studies need to implement education on this topic.

